# The Bacteriology of Every-Day Practice

**Published:** 1901-06

**Authors:** 


**The Bacteriology of Every-Day Practice.** By J. Odery Symes, M.D. Pp. 89. London: Baillière, Tindall and Cox. 1900.

As science continues to progress, it is unavoidable that those who have already passed through their student days should find themselves left behind. Their daily occupation in many cases prevents them from devoting time to keeping up their knowledge by reference to the periodicals which deal with recent researches in the various branches, and when the branch itself is a young one and full of details the difficulty is intensified. Dr. Symes' *Bacteriology of Every-Day Practice* is intended to assist those who passed through their professional curriculum before bacteriology had taken a recognised place as a subject of instruction. How well it is adapted to carry out this intention may be realised when we state that in a space of less than one hundred pages there has been found room for chapters on materials and instruments, the preparation and staining of films, general infections of the blood, including malaria, relapsing fever, and plague; suppurative processes (with special treatment of endocarditis, puerperal fever, erysipelas, tubercular suppurations, &c.), diseases of the respiratory system, enteric fever, cholera, &c. Even this does not complete the list, as space is found for paragraphs on *post-mortem* examinations from a bacteriological standpoint, serum therapeutics, lumbar puncture, and the microscopical detection of certain parasites, whilst at the end is an appendix on the microscope by Dr. D. S. Davies, which gives just the information for lack of which money is so often wasted in the purchase of an unsatisfactory microscope.

Slips there are here and there, but these are unavoidable in a first edition, and we have not anywhere noticed a mistake of a serious nature. Any criticism we would offer would be in matters of detail, such for instance as the statement on page 17 that blood films should be prepared by "squeezing" two coverglasses together, that the fixing of films is brought about by the "coagulation" of the albumin, and that "Gram covers" should be stained in anilin gentian violet for "five minutes." We think, also, that the instructions given for fixing films in the flame are insufficient. In the section on the microscope, too, we should say that the oil immersion objectives supplied by the English makers named, with the possible exception of those of Swift, are altogether inferior to the Continental glasses. English makers seem entirely unable to make a really good lens at a reasonable price, and in cases where the definition is fairly satisfactory the working distance is absurdly short. We are rather surprised not to find some mention made of the achromatic condenser. It is somewhat expensive, but for accurate work it possesses great advantages over the ordinary Abbe. But after all these are details; the more important sections of the book are excellent, and we think that the paragraphs on serum therapeutics are likely to be particularly useful in preventing the indiscriminate administration of serum in suitable and unsuitable cases alike, which has greatly tended to throw discredit upon the whole system. One great advantage of the book is its small size; larger text-books on bacteriology and on diagnosis we have in plenty. This is essentially a "handy" book, and can be read through in an hour, whilst, when the necessity arises, information upon any point can be found immediately.

